# A Prospective Evaluation of Optical Coherence Tomography in Below-the-Knee Endovascular Interventions

**DOI:** 10.1016/j.jscai.2026.105516

**Published:** 2026-08-13

**Authors:** Jun Li, Armando Vergara-Martel, Neda S. Hassani, Vladislav Zimin, Shilp Arora, John Harlock, Fadi Elias, Tej Sheth, Yulanka Castro-Dominguez, Mehdi H. Shishehbor

**Affiliations:** a University Hospitals Harrington Heart and Vascular Institute, Cleveland, Ohio; b Case Western Reserve University School of Medicine, Cleveland, Ohio; c University Hospitals Cardiovascular Phenomics Core, Cleveland, Ohio; d Tulane University Heart and Vascular Institute, New Orleans, Louisiana; e Division of Cardiology, Department of Medicine, SSM Health St. Louis University Hospital, St. Louis, Missouri; f Division of Vascular Surgery, McMaster University, Hamilton, Ontario, Canada; g Department of Medicine, McMaster University, Hamilton, Ontario, Canada

**Keywords:** below-the-knee, chronic limb-threatening ischemia, endovascular intervention, intravascular imaging, optical coherence tomography

## Abstract

**Background:**

Image-guided therapy has become the standard of care for most, if not all, coronary interventions. However, the utility of imaging, in particular, intravascular ultrasound (IVUS) and optical coherence tomography (OCT), in endovascular interventions of the lower extremities has been limited by a lack of quantitative data and evidence-based protocols. This prospective, multicenter, core-lab adjudicated study aimed to correlate IVUS, OCT, and fluoroscopic quantitative findings in the below-the-knee tibial arteries.

**Methods:**

Patients with chronic limb-threatening ischemia were prospectively recruited from 2 sites. All patients underwent predefined protocols for obtaining angiography, IVUS, and OCT. All images were analyzed by the University Hospitals Harrington HVI Cardiovascular Imaging Core Laboratory. IVUS and OCT images were compared for vessel and lesion minimum lumen diameter, average lumen diameter, area, calcium arc, calcium thickness, and cap thickness. Clinical outcomes were ascertained per standard-of-care surveillance visits and hemodynamic data for 1 year.

**Results:**

Forty-four patients were enrolled between August 2021 and June 2023. Most (92.5%) had Rutherford V to VI disease, and nearly half had chronic total occlusion. Undiluted (100%) contrast provided better image quality and longer clear image length (70.2 vs 54.7 mm, *P* = .03) compared to diluted (50%) contrast. Quantitative measurements by OCT correlated well with IVUS and angiographic analysis, though the former provides the most defined calcium analysis. Patients with increased calcium thickness in OCT analysis trended toward increased target lesion revascularization and major amputation, though with no difference in all-cause mortality.

**Conclusions:**

Optical coherence tomography of tibial arteries is feasible and correlates well with IVUS. However, its unique ability to better define tibial artery calcification burden compared to IVUS and fluoroscopy may alter treatment strategies for below-the-knee vessels in chronic limb-threatening ischemia as the landscape of therapies continues to transform.

## Introduction

A plethora of plaque modification devices (eg, atherectomy devices, specialty balloons, retractable stents, and intravascular lithotripsy) and drug-eluting therapies (eg, drug-coated balloons, drug-eluting stents [DES], and bioresorbable scaffolds) exist in contemporary endovascular therapies. Nonetheless, durable outcomes in below-the-knee (BTK) interventions remain challenging—likely owing to several unique aspects of tibial arteries, including smaller diameter vessels with long segment lesions and prevailing circumferential calcium. Angiographic interpretation of calcium remains subpar compared to intravascular imaging (IVI).^1^^,^^2^ Although intravascular ultrasound (IVUS) is reasonable for analysis in the aortoiliac and femoropopliteal segments, its inability to penetrate through calcium limits its utility in the tibial arteries.

Optical coherence tomography (OCT) provides superior spatial resolution and interpretation of calcium burden.^3^ As such, OCT may be a valuable augmentative resource to understand proper lesion preparation and mechanisms of drug delivery in BTK vessels. However, challenges in performing OCT in tibial arteries exist. We conducted a prospective study (ClinicalTrials.gov) to define OCT image protocol guidance, provide quantitative and calcium analyses in comparison to IVUS, and correlate with clinical findings in patients with chronic limb-threatening ischemia (CLTI).

## Methods

### Patient recruitment

Patients with CLTI, defined by Rutherford classification IV to VI symptoms, were recruited from 2 sites: University Hospitals Harrington Heart and Vascular Institute (HVI) and McMaster University. Patients were prospectively enrolled with a prespecified protocol for OCT and IVUS, along with traditional digital subtraction angiography. Only those with evidence of chronic kidney disease with an estimated glomerular filtration rate <30 mL/min but not on dialysis were excluded.

### Imaging technique

**Figure 1:**
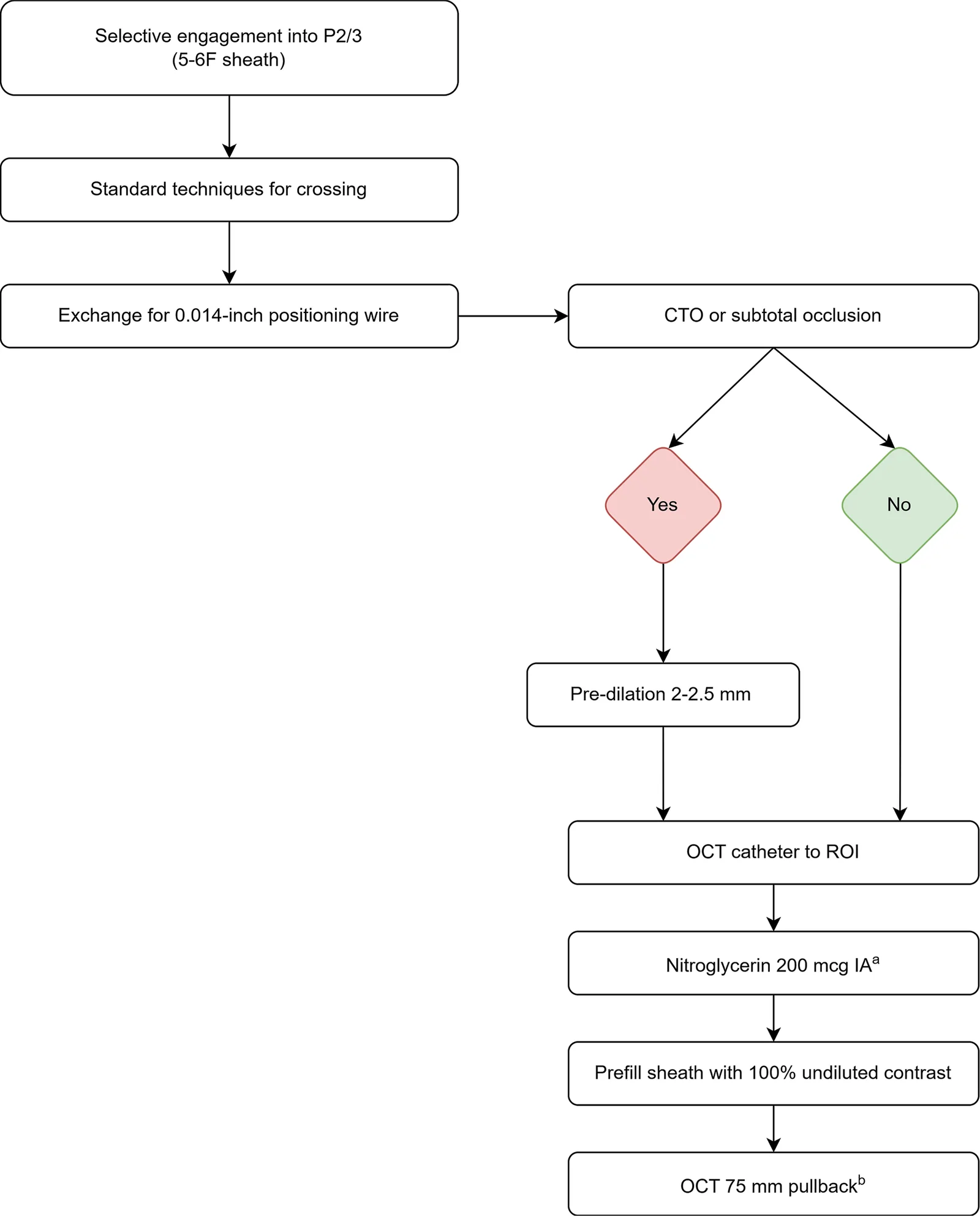
**Workflow for optimization of below-the-knee optical coherence tomography (OCT).**^a^If vasodilation is not contraindicated. ^b^Manual trigger versus automatic trigger, per operator preference. CTO, chronic total occlusion; IA, intraarterial; mcg, micrograms; ROI, region of interest.

Preceding intervention of inflow lesions (suprainguinal and/or above-knee) was performed as needed. The distal popliteal artery of the treatment limb was selectively engaged with either a 5F or 6F interventional sheath (Figure 1). Following successful crossing of the tibial artery of interest using standard techniques, we advanced a 0.014-inch guide wire. Occlusive tibial lesions were predilated with an undersized balloon (2.0-2.5 mm) to allow better opacification of the artery and visualization by OCT. Stenotic lesions were not predilated prior to imaging. Intraarterial nitroglycerin (up to 200 μg) was administered for vasodilation prior to IVI if there were no contraindications. All patients received systemic heparin for anticoagulation with a goal of an activated clotting time >250 seconds.

Preintervention OCT was performed using the Dragonfly Optis catheter (Abbott Vascular) using 2 different concentrations of Visapaque contrast media: 50% (diluted with heparinized saline) and 100% undiluted contrast. Pullback length was set to 75 mm, with an operator-triggered mechanism for initiation of pullback. Timing of pullback activation was determined by the operator when the injected contrast was visualized to approach the imaging marker.

Preintervention IVUS was performed on the same tibial segment using the 0.014-inch Eagle Eye IVUS catheter (Phillips) in patients prespecified to have concomitant IVUS interrogation. Intervention was performed per standard of care, which entails percutaneous transcatheter angioplasty as primary therapy, and bailout stent placement with DES if there is the presence of angiographic flow-limiting stenosis or dissection. Completion OCT with 100% Visapaque contrast and IVUS was performed on the same segment postintervention.

### Core laboratory analysis

Offline analyses of OCT and IVUS pullbacks were performed by University Hospitals Harrington HVI Cardiovascular Imaging Core Laboratory using the CAAS IntraVascular 2.1 software (Pie Medical Imaging). The method of analysis for each IVI modality is as described below. Fluoroscopic calcium analysis was performed offline by the University Hospitals Harrington HVI Cardiovascular Imaging Core Laboratory.

### OCT analysis protocol

Each data set was imported in raw format. The region of interest (ROI) was defined by the operator, with exclusion of frames corresponding to the interventional sheath. Reference segments were manually selected as the frame proximal and distal to the ROI and could be adjusted based on anatomic landmarks or observed artifacts. Lumen contours were automatically segmented using longitudinal reconstruction across 4 orthogonal planes, with further manual correction performed in both the longitudinal and cross-sectional views to ensure contour accuracy. Analysis was done at every 1 mm frame interval. The minimum lumen diameter (MLD), average lumen diameter (ALD), and area were derived from each segmented frame. The frame with the smallest area was designated as the minimum lumen frame (MLF). All measurements were reviewed and confirmed by 2 independent observers with adjudication in cases of discrepancy.

Clear image length (CIL) describes the cumulative OCT pullback within the target vessel, in which sufficient clearance of blood allows for visualization of the vessel wall. Derivation of cumulative CIL is calculated based on the following (Supplemental Figure S1):

1. Clear image frame, whereby the boundary between lumen and vessel wall is discernible along a continuous arc of ≥270°.
2. Clear image segment, defined as an image analysis segment (1 mm length), along which at least 1 clear image frame is present.
3. CIL is determined by the number of clear image segments within the entirety of the 75 mm pullback.
4. Calculated CIL percentage is based on CIL in millimeters divided by total image length in millimeters.

Additional CIL was obtained within the ROI, which was determined by the span of the lesion of interest along with adjoining proximal and distal reference segments, up to 5 mm on each side.

### IVUS analysis protocol

Each data set was imported in DICOM format, and the ROI was defined by the operator based on visual assessment, excluding frames corresponding to the interventional sheath or affected by artifact. Reference segments were selected as the proximal and distal frames surrounding the ROI and adjusted as needed based on anatomic landmarks or image quality. Lumen contours were generated using the software’s semiautomated segmentation, followed by manual refinement using control points in longitudinal reconstructions across 4 orthogonal planes. Contour accuracy was further verified and corrected in the cross-sectional view. Measurements were obtained at every 1 mm frame interval. The MLD, ALD, and area were calculated, with the frame with the smallest area identified as the MLF. All analyses were reviewed and confirmed by 2 independent observers with adjudication in cases of discrepancy.

### Calcium analysis protocol

Patients with cumulative and ROI CIL ≥70% were included for calcium analysis. The arc of calcium and the thickness of the overlying fibrotic cap were assessed by both OCT and IVUS at MLF for quantitative comparison. The depth of calcium was analyzed on OCT, but not feasible with IVUS because of acoustic shadowing.

In addition, we performed sequential calcium analysis on OCT, encompassing every 5 mm from the MLF, both proximally and distally (Supplemental Figure S2), with 10 additional frames and encompassing 50 mm of length analysis. Fluoroscopic assessment of the burden of calcium was analyzed using the Peripheral Artery Calcification Scoring System (PACSS)^4^ for comparison against the overall calcium burden by OCT.

### Patient clinical follow-up

Patients were followed up at intervals of 1, 3, 6, and 12 months postindex intervention with concomitant ankle-brachial index and toe-brachial index per institutional standard of care for CLTI patients. Clinical outcomes, including mortality, major amputation, wound healing, and target lesion revascularization (TLR), were obtained. Major adverse limb event (MALE) was composed of all-cause mortality, major amputation, and TLR.

### Statistical analysis

Statistical analysis was performed using SAS 9.4 software (SAS Institute). Categorical variables were presented with frequency and percentage. All continuous variables were presented with median and interquartile range. Comparison between continuous variables from preintervention to postintervention and between OCT and IVUS was performed using the Wilcoxon rank-sum test. Correlation between continuous variables was performed using Pearson’s correlation test, with the coefficient (r) used to assess the strength of association. *P* values of <.05 were considered significant.

This study was approved by the local institutional review board, and it is registered on ClinicalTrials.gov with the identifier NCT04748965. The study was supported by an unrestricted grant from Abbott Vascular. Analyses of OCT, IVUS, and angiograms were completed by the University Hospitals Harrington HVI Cardiovascular Imaging Core Laboratory. Clinical data were collected from the electronic medical record and entered into the Research Electronic Data Capture (REDCap) tool.^5^ Data safety monitoring was conducted per the institutional guidelines of the University Hospitals.

## Results

A total of 44 patients were recruited for this pilot study between August 2021 and June 2023. Patient characteristics are shown in Table 1, with a median age of 71 years and a slight male predominance (61.4%). Nearly half had prior peripheral revascularization, and most had cardiovascular comorbidities, including hypertension (84.1%), dyslipidemia (70.5%), and diabetes (70.5%). The number of patients with end-stage renal disease, either on hemodialysis or peritoneal dialysis, was 15%. The majority of patients presented with Rutherford classification V to VI symptoms (V 55%, VI 37.5%).

**Table 1:** Baseline patient characteristics in the combined US and Canadian cohorts.

Patient characteristics	N = 44
Age, y	71 (64-78)
Sex	
Male	27 (61.3%)
Female	17 (38.6%)
Race^a^	
Non-Hispanic White	35 (87.5%)
Black	3 (7.5%)
Middle Eastern	1 (2.5%)
Hispanic	1 (2.5%)
Weight, kg^a^	86 (75-97)
Height, cm^a^	172 (163-180)
Body mass index, kg/m^2,^^a^	27.1 (24.9-30.3)
Medical history	
Prior leg intervention/surgery^a^	18 (45.0%)
Coronary atherosclerosis^a^	11 (27.5%)
Hypertension	37 (84.1%)
Dyslipidemia	31 (70.5%)
Diabetes mellitus	31 (70.5%)
HFrEF (ejection fraction <50%)^a^	4 (10%)
Cerebrovascular accident/transient ischemia attack	4 (9.1%)
Atrial fibrillation^a^	9 (22.5%)
Carotid disease^a^	9 (22.5%)
Thoracic aortic aneurysm^a^	1 (2.5%)
Abdominal aortic aneurysm^a^	1 (2.5%)
Renal/mesenteric artery disease^a^	1 (2.5%)
Valvular disease^a^	1 (2.5%)
End-stage renal disease^a^	6 (15%)
Chronic obstructive pulmonary disease^a^	2 (5%)
Malignancy^a^	2 (5%)
Autoimmune disease^a^	1 (2.5%)
Smoking	
Ongoing	11 (25%)
Former	18 (40.9%)
Never	15 (34.1%)
Preceding laboratory findings^a^	
White blood cell count, ×10^9^/L	8.3 (7.3-10.2)
Hemoglobin, g/dL	12.7 (10.7-13.8)
Hematocrit, g/dL	38.6 (34.2-42.4)
Platelets, ×10^3^/dL	253 (200-338)
Blood urea nitrogen, mg/dL	20 (18-28)
Creatinine, mg/dL	1.06 (0.87-1.23)
Albumin, g/dL	3.7 (3.5-4.1)
Clinical presentation^a^	
Chronic limb-threatening ischemia	38 (95%)
Acute or subacute limb ischemia	2 (5%)
Procedural timing^a^	
Emergent (revascularization <24 h)	5 (12.5%)
Urgent (revascularization <7-14 d)	24 (60%)
Elective	11 (27.5%)
Rutherford classification^a^	
VI (major tissue loss)	15 (37.5%)
V (minor tissue loss)	22 (55%)
IV (ischemic rest pain)	3 (7.5%)

Values are median (IQR) or n (%).

HFrEF, heart failure reduced ejection fraction.

a Denotes data for the US cohort only.

Nearly half of the studied tibial arteries had chronic total occlusions (Table 2). Various crossing techniques were used, with most chronic total occlusions (13 of 19) requiring retrograde tibial access. Two patients underwent laser atherectomy for thrombotic occlusions. DES were placed in 20% of cases because of suboptimal angiographic results after primary percutaneous transcatheter angioplasty. Final balloon sizes ranged from 3 to 4 mm (median, 3.5 mm). Median volume of contrast use was 100 mL.

**Table 2:** Procedural characteristics for patients who underwent tibial artery intervention (US cohort).

Procedural characteristics	N = 40
Inflow vessel concomitant therapy	
Iliac	0
Femoropopliteal	7 (17.5%)
Imaged tibial artery	
AT	16 (40%)
TPT	
TPT only	1 (2.5%)
TPT and peroneal	6 (15%)
TPT and PT	4 (10%)
Peroneal	2 (5%)
PT	11 (27.5%)
CTO present	19 (47.5%)
CTO crossing technique	
Augmentative retrograde tibial access	13
Antegrade intimal tracking	3
Limited antegrade subintimal tracking	3
Limited retrograde subintimal tracking	4
Dissection and reentry (without reentry device)	6
Reverse CART	3
Predilation required	22 (55%)
Predilation balloon, mm	2.0 (2.0-2.5)
Atherectomy	2 (5%)
Stent used	8 (20%)
AT	2
TPT into PT	3
TPT into the peroneal	3
Final balloon size, mm	3.5 (3.0-4.0)
Total procedural time, min	129 (111-184)
Total contrast use, mL	100 (82.5-125)

Values are median (IQR) or n (%).

AT, anterior tibial; CART, controlled antegrade and retrograde tracking; CTO, chronic total occlusion; PT, posterior tibial; TPT, tibioperoneal trunk.

### OCT analysis

Quantitative OCT measurements were available in 43 preintervention and 40 postintervention patients (Table 3). In the preintervention assessment, contrast concentration did not affect the measurements of the distal reference segment. MLD at the MLF frame was approximately 1.0 mm across both contrast concentrations. The MLF ALD and area showed some variability in measurements, depending on the concentration of contrast (ALD, 1.1 vs 1.21 mm, 50% vs 100% contrast, *P* = .09; area, 0.87 vs 1.13 mm^2^, 50% vs 100% contrast, *P* = .09), but these differences remained statistically nonsignificant. Diluted contrast did significantly lower the integrity of preintervention proximal reference ALD (2.54 vs 2.91 mm, 50% vs 100% contrast, *P* = .047) and area (4.80 vs 6.62 mm^2^, 50% vs 100% contrast, *P* = .03). Postintervention, the MLF MLD increased to 1.53 mm and ALD increased to 1.97 mm in the narrowest remaining segment, despite the use of postdilation balloon sizes of 3 to 4 mm. The area improved from 1.09 to 3.17 mm^2^ (*P* < .001) at MLF on final OCT assessment in the matched-cohort comparison.

**Table 3:** Lesion and reference measurements based on OCT.

Component	Preintervention 50% (n = 43)	Preintervention 100% (n = 43)	*P* value	Postintervention 100% (n = 40)
Distal				
MLD, mm	1.48 (1.00-1.97)	1.64 (1.00-2.20)	.88	1.97 (1.56-2.45)
ALD, mm	1.60 (1.01-2.48)	1.83 (1.05-2.41)	.54	2.34 (1.8-2.7)
Area, mm^2^	2.0 (0.80-4.81)	2.59 (0.86-4.57)	.50	4.2 (2.49-5.60)
MLF				
MLD, mm	0.96 (0.89-1.12)	1.01 (0.92-1.28)	.13	1.53 (1.07-1.93)
ALD, mm	1.1 (0.99-1.43)	1.21 (1.00-1.57)	.09	1.97 (1.6-2.3)
Area, mm^2^	0.87 (0.78-1.63)	1.13 (0.80-1.96)	.09	3.12 (1.96-4.29)
Proximal				
MLD, mm	2.24 (1.36-3.13)	2.66 (1.97-3.51)	.05	2.72 (2.34-3.26)
ALD, mm	2.54 (1.56-3.32)	2.91 (2.22-3.86)	.047	3.0 (2.57-3.70)
Area, mm^2^	4.80 (1.77-8.52)	6.62 (3.72-11.53)	.03	7.15 (5.12-11.24)

Values are median (IQR).

Preintervention data were collected using 50% and 100% contrast in the US and Canadian cohorts; *P* values compare these dilutions. Postintervention data (100% contrast) are from the US cohort only.

Distal and proximal refer to the respective reference frames. MLF is determined by the frame containing the smallest lumen area.

ALD, average lumen diameter; MLD, minimum lumen diameter; MLF, minimum lumen frame; OCT, optical coherence tomography.

Clear image length was statistically higher in preintervention OCT pullbacks utilizing undiluted contrast (54.7 vs 70.2 mm, 50% vs 100% contrast, *P* = .03; Supplemental Table S1). Within the ROI, there was a trend toward improved CIL in the 100% contrast cohort, but this did not reach statistical significance. In patients with matched preintervention and postintervention pullbacks (Supplemental Table S2), postintervention cumulative CIL was higher (99 vs 88%, postintervention vs preintervention, *P* = .02). The number of patients achieving >90% CIL rose from 18 (46.2%) preintervention to 30 (76.9%) postintervention (*P* = .005).

### OCT versus IVUS quantitative analysis

**Figure 2:**
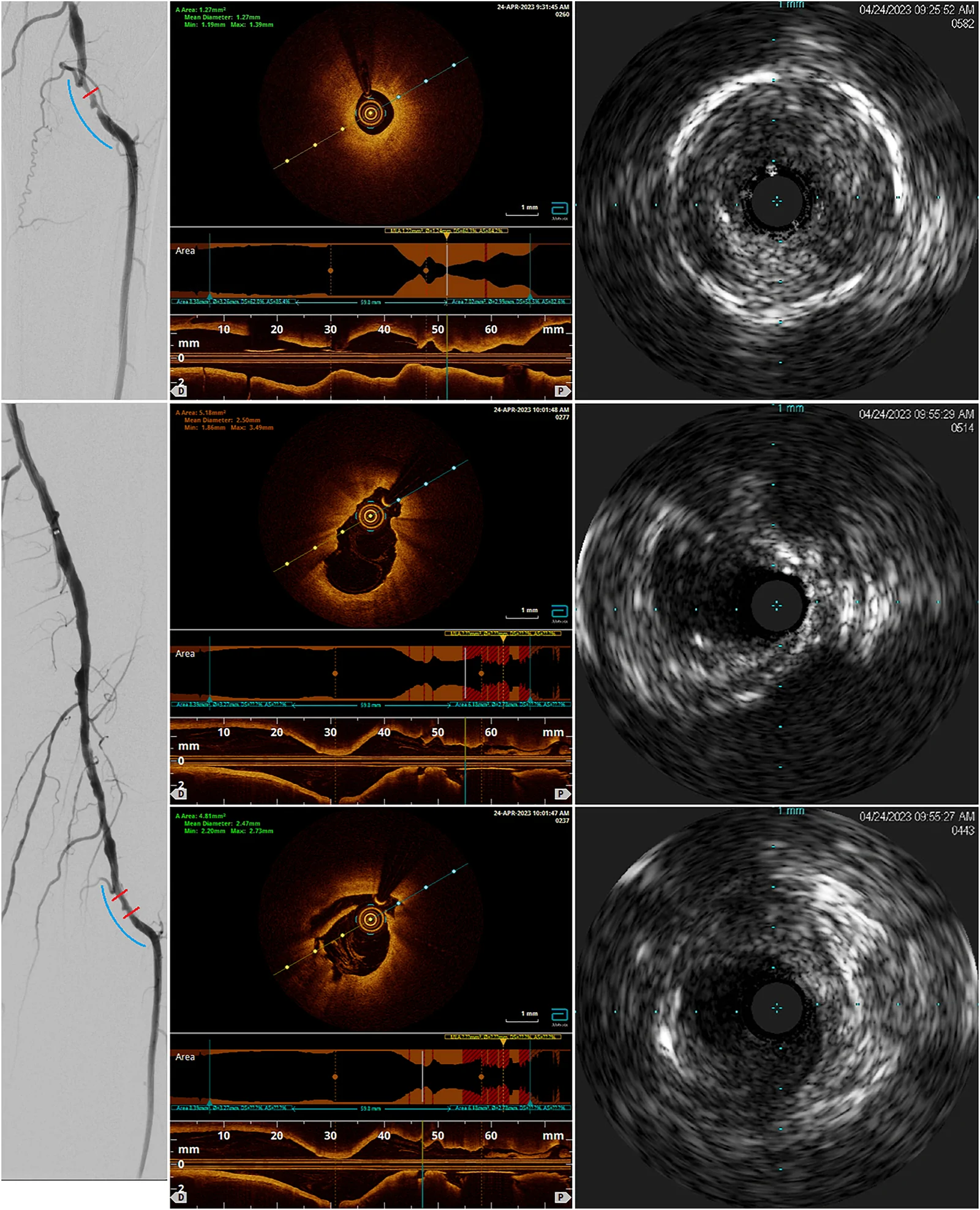
**A patient with severe proximal anterior tibialdisease and occlusion of the tibioperoneal trunk, posterior tibial, and peroneal artery.** Preangiograms and postangiograms with corresponding optical coherence tomography (OCT) and intravascular ultrasound (IVUS) frames are shown. The region of interest is marked by a blue arc; red lines indicate OCT and IVUS cross-sections. In the preintervention image (top panel), the lesion appears highly fibrotic on OCT and heterogeneous on IVUS. IVUS shows adventitial calcium not clearly visible on OCT because of attenuation from fibrolipid-rich plaque. Postintervention OCT shows reasonable lumen gain in proximal anterior tibial artery (mid panel), but a dissection flap is seen distally (bottom panel). IVUS visualization of the lumen and flap is less distinct.

Preintervention matched IVUS was available for analysis in 34 patients and postintervention in 32 patients; a total of 29 patients had both preintervention and postintervention IVUS and OCT for comparison. Figure 2 demonstrates a sample case with both preintervention and postintervention IVI. Analysis of the distal reference, minimum lumen, and proximal reference frames for both preintervention and postintervention yielded higher measurements with IVUS than with OCT (Table 4). Correlation testing showed reasonable agreement between the 2 modalities in preintervention, which improved in postintervention (Supplemental Figures S3 and S4). Delta change from preintervention to postintervention was greater on OCT for MLD and area at the MLF than IVUS (MLD: 0.43 vs 0.18 mm; area: 1.64 vs 1.03 mm^2^; *P* = .002; Table 4).

**Table 4:** Comparison of OCT and IVUS in patients with matched preintervention and postintervention pullbacks.

Component	OCT	IVUS	*P* value
Preintervention	n = 34	n = 34	
Distal			
MLD, mm	1.5 (1.0-2.0)	1.9 (1.6-2.4)	.003
ALD, mm	1.7 (1.0-2.4)	2.5 (1.8-2.6)	.004
Area, mm^2^	2.2 (0.9-4.3)	3.5 (2.5-5.1)	.005
MLF			
MLD, mm	1.0 (0.9-1.2)	1.7 (1.6-1.8)	<.001
ALD, mm	1.2 (1.0-1.5)	1.8 (1.7-2.0)	<.001
Area, mm^2^	1.1 (0.8-1.7)	2.7 (2.3-3.4)	<.001
Proximal			
MLD, mm	2.6 (1.8-3.4)	3.3 (2.6-3.9)	.03
ALD, mm	2.8 (2.1-3.8)	3.6 (2.9-4.2)	.04
Area, mm^2^	6.2 (3.4-11.1)	9.9 (6.5-13.8)	.03
Postintervention	n = 32	n = 32	
Distal			
MLD, mm	1.9 (1.6-2.4)	2.3 (2.0-2.6)	.01
ALD, mm	2.3 (1.8-2.7)	2.5 (2.2-2.8)	.09
Area, mm^2^	4.0 (2.4-5.6)	4.8 (3.7-6.0)	.08
MLF			
MLD, mm	1.5 (1.1-1.9)	1.9 (1.7-2.3)	<.001
ALD, mm	2.0 (1.6-2.3)	2.1 (1.9-2.5)	.025
Area, mm^2^	3.2 (2.0-4.1)	3.6 (2.8-5.1)	.03
Proximal			
MLD, mm	2.6 (2.3-3.0)	3.2 (2.6-3.8)	.02
ALD, mm	2.9 (2.6-3.7)	3.5 (2.8-4.0)	.05
Area, mm^2^	6.6 (5.1-11.0)	8.9 (6.4-12.5)	.049
Change from preintervention to postintervention	n = 29	n = 29	
Distal			
MLD, mm	0.50 (0.02-0.90)	0.024 (0.0-0.53)	.09
ALD, mm	0.67 (0.0-0.98)	0.31 (–0.0 to 0.62)	.02
Area, mm^2^	1.7 (0.0-3.07)	1.3 (0.0-2.02)	.05
MLF			
MLD, mm	0.43 (0.23-0.86)	0.18 (0.05-0.48)	.002
ALD, mm	0.69 (0.39-0.95)	0.11 (0.32-0.55)	.001
Area, mm^2^	1.64 (0.85-3.02)	1.03 (0.32-2.1)	.002
Proximal			
MLD mm	0.13 (–0.27 to 0.54)	0.05 (–0.23 to 0.29)	.35
ALD, mm	0.10 (–0.24 to 0.47)	0.07 (–0.21 to 0.36)	.53
Area, mm^2^	0.58 (–0.74 to 2.15)	0.20 (–1.38 to 1.96)	.52
Calcium and cap	n = 20	n = 20	
Calcium thickness, mm	0.49 (0.26-0.80)	NA	—
Calcium arc, °	130 (90-183)	163 (94-273)	.26
Cap thickness, mm	0.23 (0.09-0.33)	0.12 (0.07-0.25)	.27

Values are median (IQR).

Change in preintervention to postintervention reflects the delta in patients with both pullbacks. Calcium and cap assessments were limited to those with calcium at the MLF. Calcium thickness was not measured on IVUS because of acoustic shadowing.

ALD, average lumen diameter; IVUS, intravascular ultrasound; MLD, minimum lumen diameter; MLF, minimum lumen frame; NA, not applicable; OCT, optical coherence tomography.

### OCT versus IVUS calcium analysis

**Figure 3:**
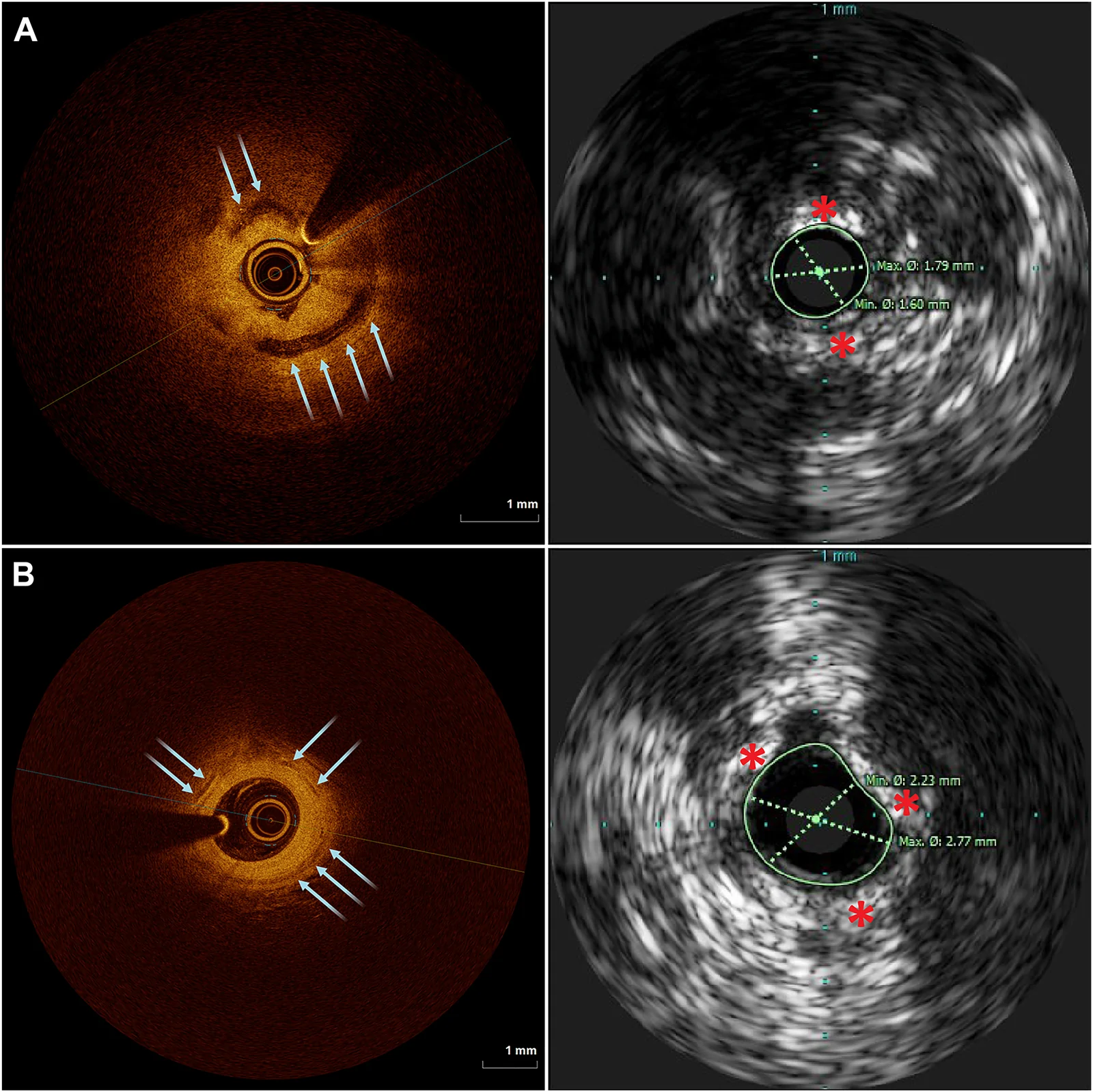
**Examples oftwo different subjectswithcorresponding optical coherence tomography and intravascular ultrasound MLF with calcium.** Blue arrows denote calcium noted on optical coherence tomography, and red asterisks mark calcium on intravascular ultrasound.

Calcium (Figure 3) was analyzed at the MLF on preintervention OCT and IVUS pullbacks. Six preintervention pullbacks were excluded because of CIL <70%, and 2 patients were excluded because of a lack of concomitant IVUS images for comparison. One patient was excluded because of a lack of preintervention OCT. Of the remaining 31 patients, 11 showed no calcium on OCT at MLF. A total of 20 patients (64.5% of the analyzed 31 patients) were deemed to have calcium present at MLF and underwent analysis (Table 4). This showed a median calcium arc of 130° by OCT and 163° by IVUS (*P* = .26), with a cap thickness of 0.24 mm by OCT and 0.12 mm by IVUS (*P* = .27). Calcium thickness was found to be 0.49 mm by OCT.

### OCT versus fluoroscopic calcium analysis

In the same 31 patients, sequential calcium analysis (Supplmental Figure S2) was performed of the 50 mm flanking the MLF (25 mm pre- and 25 mm post-MLF), constituting 11 frames in total per patient. The median arc of all analyzed frames was 138°, with a median calcium thickness of 0.55 mm and a median cap thickness of 0.21 mm.

At the lesion of interest, PACSS analysis showed a bimodal distribution of calcium (Supplemental Figure S5), with most patients falling within grade 3 to 4. When compared to OCT, higher grades of PACSS correlated with higher calcium arc, increased calcium thickness, and a greater number of OCT frames with calcium present within 50 mm of the MLF (Supplemental Figure S5, Table 5), although these were not statistically significant.

**Table 5:** Comparison of OCT and angiographic calcium analysis.

PACSS score	≤2 (n = 14)	3 (n = 6)	4 (n = 11)	*P* value
Number of OCT frames with calcium	4.5 (2-6)	6.5 (4-9)	8 (3-11)	.19
Calcium thickness at MLF, mm	0.06 (0.0-0.50)	0.11 (0.0-0.23)	0.47 (0.21-0.80)	.18
Maximal calcium arc, °	23.5 (0-108)	38 (0-143)	134 (70-199)	.09

Values are median (IQR).

Angiographic PACSS grading (≤2, 3, or 4) and its relationship with the number of total OCT frames with calcium, as examined on the sequential calcium analysis (maximum number of possible frames 11), calcium thickness at MLF, and maximal arc of calcium.

MLF, minimum lumen frame; OCT, optical coherence tomography; PACSS, Peripheral Artery Calcification Scoring System.

### Clinical outcomes

Follow-up at 1 year showed a low major amputation rate (5%) and a median time-to-wound healing of 137 days. Wound healing was successful in 72.5% of patients at the conclusion of the 1-year follow-up. However, all-cause mortality was 15%, and TLR was 12.5%, resulting in a combined MALE of 27.5% at 1 year (Supplemental Table S3).

**Figure 4:**
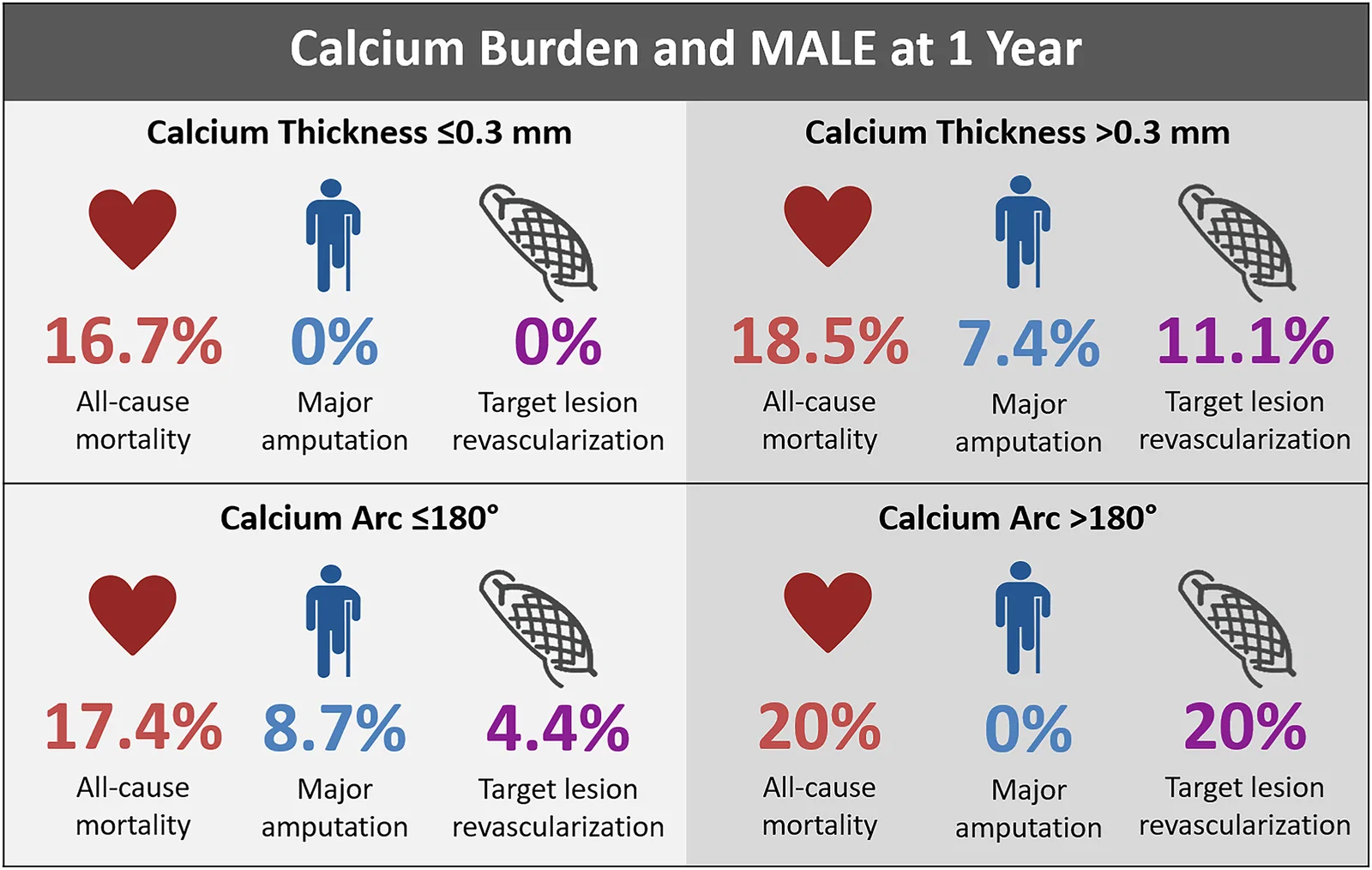
**One-year clinical major adverse limb event (MALE) outcomes based on calcium thickness and arc.** The outcomes did not reach statistical significance.

Evaluation of OCT-based calcium burden and correlation to MALE was performed in the 33 patients with preintervention OCT who had CIL ≥70%. No significant difference was noted (Figure 4, Supplemental Table S4).

## Discussion

This is the first prospective, multicenter, core-lab adjudicated study evaluating the use of OCT, IVUS, and fluoroscopic correlation in BTK tibial vessels. Prior smaller studies have demonstrated early clinical experience and the feasibility of performing IVUS and OCT in the tibial arteries.^6^, ^7^, ^8^ This study builds upon those foundational efforts and describes the best practices and optimization techniques for frequency-domain OCT imaging of the tibial arteries. Our results demonstrate that OCT provides excellent spatial resolution and can identify calcium location, thickness, and extent much more clearly than IVUS or fluoroscopy, which may impact novel BTK therapeutics. We have also shown that the use of either 50% diluted and 100% undiluted contrast media is feasible for blood clearance during preintervention BTK OCT imaging, but the fidelity of imaging in the proximal ROI is better with undiluted contrast. The improved visualization is likely because of the higher viscosity of undiluted contrast, which allows more effective and prolonged blood displacement for OCT imaging.

Various flush media for the efficacy of blood clearance for OCT imaging have previously been evaluated in the superficial femoral artery (SFA) in the OPTical Imaging Measurement of Intravascular Solution Efficacy (OPTIMISE) study.^9^ In the SFA study, the types of media tested included contrast, heparinized saline, dextran, and carbon dioxide, with image quality assessed by clear imaging field criteria similar to those used in the current study. Although the prior analysis suggested equivalent imaging quality with the first 3 types of media, the volume required to achieve adequate imaging differed strikingly (contrast mean 12 mL, heparinized saline 20 mL, and dextran 19 mL).^9^ In BTK imaging, dextran often causes significant calf and foot discomfort, leading to its exclusion from the current study. Furthermore, in clinical practice, it is more practical to acquire simultaneous angiographic assessment along with an OCT pullback. As such, our trial focused on comparing half-strength contrast with undiluted contrast, rather than evaluating noncontrast options.

All of the OCT acquisitions in this study required <10 mL of volume per run. Despite multiple OCT pullbacks per patient as part of the study protocol, our median volume of contrast used in this trial remained only 100 mL. Strategies to spare contrast use in endovascular procedures have been described previously,^10^ using a “bolus-and-chase” technique with 1:4 contrast-to-saline dilution with digital subtraction angiography imaging. With this method, most traditional endovascular interventions require <50 mL of contrast, permitting more liberal use of undiluted contrast for OCT acquisitions while still maintaining an overall low contrast volume administration.

As anticipated, postintervention CIL surpassed preintervention undiluted contrast CIL, in both the cumulative evaluation and within the defined ROI. Adequate vessel dilation prior to the final OCT imaging enhances imaging quality by facilitating efficient contrast flow and more complete blood displacement. Surprisingly, however, despite the use of a median balloon size of 3.5 mm during our final dilation, the MLD within the MLF remained 1.53 mm in the postintervention assessment by OCT. This finding indicates notable vessel recoil occurring even within minutes after balloon dilation, which may have implications on procedural optimization in terms of acute- and long-term luminal gain. This study was not designed to evaluate plaque modification techniques, but future endeavors may favor the use of OCT to understand the effects on lumen gain compared to traditional balloon angioplasty.

In our analysis of IVUS compared to OCT in BTK tibial vessels, significant differences in diameter and area measurements were observed between the 2 modalities in both preintervention and postintervention cohorts. Nonetheless, correlation testing showed reasonable agreement between the 2 types of IVI. The divergence between OCT and IVUS in BTK vessel sizing is consistent with prior coronary studies that have shown discrepancies between these 2 imaging modalities.^11^^,^^12^ Differences in coronary analysis have been attributed to the methodology by which measurements are ascertained: OCT utilizes a lumen-based method, whereas IVUS sizing is derived from the external elastic lamina.^3^ In coronary interventions, the sizing strategy for the DES diameter (ie, downsizing or upsizing by a half millimeter) differs based on the IVI technique.^13^ Future studies in BTK IVI should explore the applicability of this particular coronary-based stent sizing strategy in peripheral interventions and evaluate its effectiveness in maintaining long-term lesion patency.

For exploratory purposes, we compared the change in diameter and area preintervention and postintervention in patients with both OCT and IVUS available. Within the MLF, changes were more pronounced on OCT than on IVUS, as reflected by larger deltas in MLD and area, and to a lesser extent, in the distal reference ALD. This greater delta observed on OCT may be partially attributed to improved blood clearance following intervention, which aligns with the improvement in CIL postintervention.

Recognizing the preponderance of calcium deposition in BTK vessels may change the landscape of therapies in the management of CLTI patients with tibial disease (Central Illustration). Approximately two-thirds of analyzed patients had evidence of BTK calcium, whereas the remaining one-third demonstrated no calcium by either OCT or IVUS. Notably, 1 patient exhibited deep, adventitial calcium on IVUS that was not visualized on OCT because of attenuation of the near-infrared light signal from overlying fibrolipid-rich plaque (Figure 2). This underscores a known limitation of OCT: although it provides high-resolution calcium thickness assessment, it is susceptible to signal loss in certain tissue types.^13^^,^^14^ Conversely, IVUS is less affected by soft tissue attenuation but cannot measure calcium thickness.^14^ Akin to coronary imaging,^15^ the arc of calcium was larger on IVUS, potentially because of blooming artifact, but this did not reach statistical significance in our analysis.

**Central Illustration:**
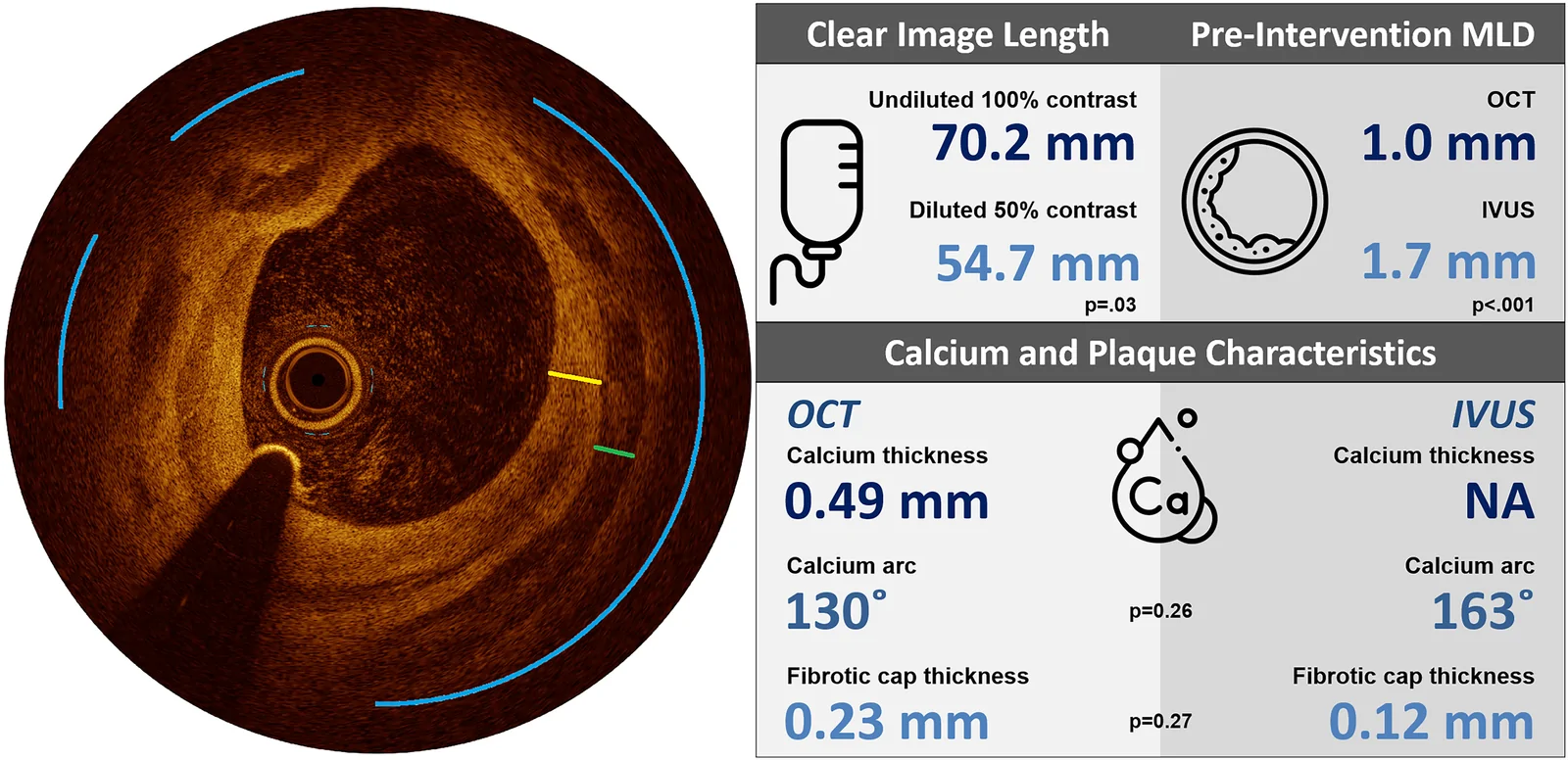
Calcium analysis by optical coherence tomography (OCT) is shown in the left panel, with blue denoting the arc of calcium, which is incontiguous in this example. The fibrotic cap thickness and calcium thickness are designated by yellow and green lines, respectively. Clear image length analysis is congruent with undiluted contrast, providing better imaging quality than diluted, 50% contrast on OCT imaging (*P* = .028). Preintervention minimum lumen diameter (MLD) was lower by OCT compared to intravascular ultrasound (IVUS) (*P* < .001), likely owing to mechanistic differences in image acquisition in the 2 modalities, whereby the former measures intraluminal diameter and the latter measures external elastic lamina. Calcium and fibrotic plaque characteristics were not different between the 2 intravascular imaging modalities, though OCT is capable of capturing calcium thickness, whereas IVUS is unable to penetrate through calcium to measure the depth of involvement. NA, not applicable.

Although the median arc of calcium calculated in the OCT sequential analysis in our study occupied less than 2 quadrants, the median calcium depth was 0.55 mm based on multiframe analysis. Based on a recently revised coronary OCT-derived calcium score, calcium occupancy of arc >270°, thickness >0.3 mm, and length >3 mm^16^ are correlated with stent underexpansion. From a clinical perspective in BTK interventions, the barrier of calcium may obviate the effects of antiproliferatives, as previously shown in the SFA territory,^17^ and may account for previous failed drug-coated balloon studies in the BTK space.^18^^,^^19^ Furthermore, as bioresorbable scaffolds become more widely adopted into clinical practice, attention to calcium-related scaffold underexpansion will be vital, as it may contribute to target vessel thrombosis.^20^ Lastly, the ability to distinguish between luminal, medial, and adventitial calcium may hold clinical implications in BTK interventions. Although our study is underpowered to fully evaluate the burden of calcium on MALE, we did note nonsignificant trends toward elevated rates of TLR with increasing calcium thickness and arc. Further investigation is needed into effective plaque modification and calcium disruption to improve long-term CLTI outcomes, and the adjunct of OCT may help tailor treatment strategies.

Common barriers to the adaptation of any IVI in endovascular revascularization include incremental increases in upfront procedural cost and time. However, emerging evidence—particularly with IVUS—demonstrates improved clinical outcomes, including major amputation^21^ and TLR,^22^ rendering IVI a cost-effective strategy over the full episode of care. Although OCT remains off-label in the peripheral vasculature, its adaptation into clinical practice may additionally be hindered by perceived contrast burden and operator familiarity. However, our study and prior experience^10^ have demonstrated strategies to minimize contrast exposure for patients; nonetheless, OCT with undiluted contrast should be avoided in those with advanced chronic kidney disease (eg, estimated glomerular filtration rate <30 mL/min). As for operator comfort with OCT, recent advances in OCT software have streamlined image acquisition and interpretation, reducing the learning curve for novice users.

Finally, our study reflects a representative cohort of CLTI patients, with 92.5% with Rutherford classification V to VI symptoms. Despite the advanced nature of their disease, the majority of patients achieved complete wound healing within a year, with a median healing time of 4.5 months. However, even with encouraging wound healing outcomes and adherence to optimal medical therapy for cardiovascular risk reduction, the all-cause mortality rate remained approximately 15% at 1 year, which is consistent with the lower range reported in existing CLTI mortality data.^23^ Improving mortality outcomes in this high-risk patient population remains a crucial and ongoing challenge for the cardiovascular community.

### Limitations

Some limitations exist in our current study. As the study commenced before the bioresorbable scaffold was FDA approved, the use of DES was reserved for those with angiographically insufficient results (ie, dissections with diminished distal flow), as opposed to direction from OCT. The changing landscape of technologies in BTK interventions will alter future treatment algorithms based on OCT.

This is the largest trial to date evaluating frequency-domain OCT, but the number of included patients is still relatively small. Furthermore, this was a single-cohort study in which all patients underwent OCT imaging. Future investigations will benefit from randomized comparisons, including patients with and without OCT, as well as across different IVI modalities, to better understand the respective impact on treatment strategies and clinical outcomes.

## Conclusion

Imaging of tibial vessels is feasible with OCT, with better imaging integrity utilizing undiluted contrast for blood clearance. Optimization techniques for image visualization, including selective engagement of the sheath into the distal popliteal segment and predilation of occlusions, allow better visualization of the vessel wall. Further evaluation is needed to assess the utility of OCT to help optimize tibial interventions and improve the durability of revascularization.

## CRediT authorship contribution statement

**Jun Li:** Writing – review & editing, Writing – original draft, Visualization, Validation, Supervision, Resources, Project administration, Methodology, Investigation, Funding acquisition, Data curation, Conceptualization. **Armando Vergara-Martel:** Software, Formal analysis. **Neda S. Hassani:** Software, Formal analysis. **Vladislav Zimin:** Software, Formal analysis. **Shilp Arora:** Writing – original draft, Validation, Formal analysis, Data curation. **John Harlock:** Methodology, Investigation, Data curation. **Fadi Elias:** Project administration, Methodology, Data curation, Conceptualization. **Tej Sheth:** Project administration, Methodology, Funding acquisition, Conceptualization. **Yulanka Castro-Dominguez:** Writing – original draft, Data curation. **Mehdi H. Shishehbor:** Writing – original draft, Methodology, Investigation.

## Declaration of competing interest

Jun Li is a consultant for Abbott Vascular, Boston Scientific, Inari Medical, Inquis Medical, and Medtronic. John Harlock reports speaker honorarium for Cook Medical and Boston Scientific, and is on the medical advisory board for Boston Scientific and Abbott. Fadi Elias reports honorarium for Cook Medical and Boston Scientific. Tej Sheth reports honorarium and research grants from Abbott Vascular and Edwards Lifesciences. Yulanka Castro-Dominguez is a consultant for Boston Scientific and Medtronic. Mehdi H. Shishehbor is on the global advisory board for Abbott Vascular, Medtronic, Terumo, Phillips, Boston Scientific, ANT, and Inquis Medical. The remaining co-authors have no disclosures.

## Footnotes

To access the supplementary material accompanying this article, visit the online version of the *Journal of the Society for Cardiovascular Angiography & Interventions* at 10.1016/j.jscai.2026.105516.
