# Supplementary material for: A Prospective Evaluation of Optical Coherence Tomography in Below-the-Knee Endovascular Interventions
Source: J Soc Cardiovasc Angiogr Interv. 2026 Aug 13;5(9):105516. doi: 10.1016/j.jscai.2026.105516 (PMC13591525; doi:10.1016/j.jscai.2026.105516)
Supplement: Supplementary file 1 [file mmc1.docx]

**Supplemental Figure S1.** Schematic of analysis for CIL on OCT. A clear image frame is defined by ≥270° of visible lumen-to-wall interface. A clear image segment requires ≥1 clear frame within a 1 mm span. Cumulative CIL is the total length of clear segments over the 75 mm pullback. In the example shown on the bottom panel, 6 mm are inadequately visualized, resulting in 69 mm (92%) CIL.

**Supplemental Figure S2.** Schematic for sequential calcium analysis on OCT. Starting from the MLF, frames are analyzed every 5 mm both proximally and distally. Including the MLF, a total of 11 frames is included in the totality of analysis along a 50 mm segment. Patients with pre-intervention OCT with CIL ≥70% were included in the sequential calcium analysis.

**Supplemental Table S1.** Comparison of pre-intervention OCT pullback CIL with either 50% or 100% contrast. Analysis was performed of the cumulative CIL (entire 75 mm run) and ROI (lesion-specific analysis).

|  | **Pre-Intervention 50%** | **Pre-Intervention 100%** | **p-value** |
| --- | --- | --- | --- |
| Total patients | 43 | 43 |  |
| Cumulative CIL |  |  |  |
| Total image length in frames | 375 (375-375) | 375 (375-375) | .999 |
| Total image length in mm | 75 (75-75) | 75 (75-75) | .999 |
| CIL in frames | 273.5 (203-374) | 351 (271-374) | .02 |
| CIL in mm | 54.7 (40.6-74.8) | 70.2 (54.2-74.8) | .03 |
| CIL (%) | 72.2 (52.8-99.7) | 93.6 (72.3-99.7) | .047 |
| ROI CIL |  |  |  |
| Total ROI in frames | 375 (210-375) | 327 (147-375) | .54 |
| Total ROI in mm | 75 (42-75) | 65 (29-75) | .54 |
| Clear ROI in frames | 207.5 (106-316) | 245 (135-327) | .34 |
| Clear ROI in mm | 41.5 (21.2-63.2) | 48.6 (27.0 - 65.4) | .36 |
| Clear ROI (%) | 86.7 (39.5-100) | 99.7 (73.6-100) | .10 |
| Number of studies with CIL >90% |  |  |  |
| Cumulative CIL >90% | 16 (37.2%) | 22 (51.2%) | .19 |
| ROI CIL >90% | 20 (46.5%) | 28 (65.1%) | .08 |

CIL, clear image length; ROI, region of interest.

**Supplemental Table S2.** Comparison of pre- and post-intervention OCT pullback CIL with 100% contrast. Analysis was performed of the cumulative CIL (entire 75 mm run) and ROI.

|  | **Pre-Intervention 100%** | **Post-Intervention 100%** | **p-value** |
| --- | --- | --- | --- |
| Total patients | 39 | 39 |  |
| Cumulative CIL |  |  |  |
| Total image length in frames | 375 (375-375) | 375 (375-375) | .999 |
| Total image length in mm | 75 (75-75) | 75 (75-75) | .999 |
| CIL in frames | 330 (263-374) | 374 (340-374) | .009 |
| CIL in mm | 66 (52-74.8) | 74.8 (68-74.8) | .02 |
| CIL (%) | 88 (70-99.7) | 99 (91-99.7) | .02 |
| ROI CIL |  |  |  |
| Total ROI in frames | 318 (147-375) | 340 (159-375) | .16 |
| Total ROI in mm | 63.6 (29.4-75.0) | 68.0 (31.8-75.0) | .13 |
| Clear ROI in frames | 243 (135-305) | 287 (148-341) | .001 |
| Clear ROI in mm | 47.6 (27-61) | 55.8 (29.6-68.2) | .007 |
| Clear ROI (%) | 1.00 (0.72-1.00) | 1.00 (0.95-1.00) | .04 |
| Number of studies with CIL >90% |  |  |  |
| Cumulative CIL >90% | 18 (46.2%) | 30 (76.9%) | .005 |
| ROI CIL >90% | 24 (61.5%) | 32 (85.1%) | .04 |

CIL, clear image length; ROI, region of interest.

**Supplemental Figure S3.** Pre-intervention correlation of IVUS and OCT measurements.

MLD, minimum lumen diameter; ALD, average lumen diameter; MLF, minimum lumen frame.

**Supplemental Figure S4**. Post-intervention correlation of IVUS and OCT measurements.

MLD, minimum lumen diameter; ALD, average lumen diameter; MLF, minimum lumen frame.

**Supplemental Figure S5.** Graphic comparison of patients with available sequential calcium analysis on OCT versus angiographic PACSS. (A) Distribution of patients analyzed based on PACSS (range 0-4). (B) Patients with PACSS grading (≤2, 3, or 4) compared to the number of frames with calcium present on sequential analysis on OCT. (C) Patients with PACSS grading (≤2, 3, or 4) compared to maximal arc of calcium by OCT.

PACSS, Peripheral Artery Calcification Scoring System; OCT, optical coherence tomography.

**Supplemental Table S3.** Patient outcomes at 1 year follow up in the US cohort.

| **Outcomes** | **n** |
| --- | --- |
| Total patients | 40 |
| MALE | 11 (27.5%) |
| Mortality | 6 (15.0%) |
| Major amputation | 2 (5.0%) |
| Target lesion revascularization | 5 (12.5%) |
| Successful wound healing | 29 (72.5%) |
| Median time to wound healing (days) | 137 (94-261) |

MALE, major adverse limb event.

**Supplemental Table S4.** Clinical outcomes at 1 year based on calcium thickness and arc as analyzed on OCT.

|  | **N** | **Calcium thickness** | | **p-value** | **Calcium arc** | | **p-value** |
| --- | --- | --- | --- | --- | --- | --- | --- |
|  |  | **≤0.3 mm** | **>0.3 mm** |  | **≤180**° | **>180**° |  |
| Patients | 33 | 6 | 27 |  | 23 | 10 |  |
| MALE | 9 (27.3%) | 1 (16.7%) | 8 (29.6%) | .35 | 6 (26.1%) | 3 (10%) | .31 |
| Mortality | 6 (18.2%) | 1 (16.7%) | 5 (18.5%) | .92 | 4/23 (17.4%) | 2/10 (20%) | .86 |
| Major amputation | 2 (6.1%) | 0 (0%) | 2 (7.4%) | .49 | 2/23 (8.7%) | 0/10 (0%) | .48 |
| TLR | 3 (9.1%) | 0 (0%) | 3 (11.1%) | .54 | 1/23 (4.4%) | 2/10 (20%) | .15 |
| Wound healing | 24 (72.7%) | 4 (66.7%) | 20 (74.1%) | .71 | 17/23 (73.9%) | 7/10 (70%) | .82 |

mm, millimeter; MALE, major adverse limb event; TLR, target lesion revascularization.
